# Structural Insights into the MMACHC-MMADHC Protein Complex Involved in Vitamin B_12_ Trafficking*

**DOI:** 10.1074/jbc.M115.683268

**Published:** 2015-10-19

**Authors:** D. Sean Froese, Jolanta Kopec, Fiona Fitzpatrick, Marion Schuller, Thomas J. McCorvie, Rod Chalk, Tanja Plessl, Victoria Fettelschoss, Brian Fowler, Matthias R. Baumgartner, Wyatt W. Yue

**Affiliations:** From the ‡ Division of Metabolism and Children's Research Center, University Children's, Hospital, CH-8032 Zurich, Switzerland,; § radiz – Rare Disease Initiative Zurich, Clinical Research Priority Program for Rare Diseases, University of Zurich, CH-8032 Zurich, Switzerland,; the ¶ Structural Genomics Consortium, Nuffield Department of Clinical Medicine, University of Oxford, Oxford OX3 7DQ, United Kingdom, and; the ‖ Zurich Center for Integrative Human Physiology, University of Zurich, 8057 Zurich, Switzerland

**Keywords:** crystal structure, metabolic disease, protein-protein interaction, site-directed mutagenesis, small-angle x-ray scattering (SAXS), nitroreductase fold, vitamin B12

## Abstract

Conversion of vitamin B_12_ (cobalamin, Cbl) into the cofactor forms methyl-Cbl (MeCbl) and adenosyl-Cbl (AdoCbl) is required for the function of two crucial enzymes, mitochondrial methylmalonyl-CoA mutase and cytosolic methionine synthase, respectively. The intracellular proteins MMACHC and MMADHC play important roles in processing and targeting the Cbl cofactor to its destination enzymes, and recent evidence suggests that they may interact while performing these essential trafficking functions. To better understand the molecular basis of this interaction, we have mapped the crucial protein regions required, indicate that Cbl is likely processed by MMACHC prior to interaction with MMADHC, and identify patient mutations on both proteins that interfere with complex formation, via different mechanisms. We further report the crystal structure of the MMADHC C-terminal region at 2.2 Å resolution, revealing a modified nitroreductase fold with surprising homology to MMACHC despite their poor sequence conservation. Because MMADHC demonstrates no known enzymatic activity, we propose it as the first protein known to repurpose the nitroreductase fold solely for protein-protein interaction. Using small angle x-ray scattering, we reveal the MMACHC-MMADHC complex as a 1:1 heterodimer and provide a structural model of this interaction, where the interaction region overlaps with the MMACHC-Cbl binding site. Together, our findings provide novel structural evidence and mechanistic insight into an essential biological process, whereby an intracellular “trafficking chaperone” highly specific for a trace element cofactor functions via protein-protein interaction, which is disrupted by inherited disease mutations.

## Teaser

**Background:** Two intracellular proteins, MMACHC and MMADHC, functionally interact for cobalamin trafficking.

**Results:** MMADHC crystal structure reveals protein-interacting regions and unexpected homology to MMACHC; mutations on either protein interfere with complex formation via different mechanisms.

**Conclusion:** Complex formation likely depends on prior cobalamin processing and can be broken by disease mutations.

**Significance:** MMACHC-MMADHC heterodimerization forms the essential trafficking chaperone delivering cobalamin to client enzymes.

## Introduction

Vitamin B_12_ (referred hereafter as cobalamin, Cbl)^5^ is an essential cofactor for two human enzymes: cytosolic methionine synthase (MS, EC 2.1.1.13), which requires methyl-Cbl (MeCbl), and mitochondrial methylmalonyl-CoA mutase (MUT, EC 5.4.99.2), which requires adenosyl-Cbl (AdoCbl) (1). An intracellular pathway of at least seven human gene loci has evolved, encoding proteins responsible for the transport, processing and delivery of the appropriate Cbl form via a cytosol-targeted route to MS, and a mitochondrion-targeted route to MUT (2, 3). Mutations in any of the seven genes, depending on its relative position within the pathway, can result in three broad disease phenotypes collectively referred to as “intracellular Cbl disorders” (2, 4). They include (i) homocystinuria (HC) associated with reduced MeCbl production and MS deficiency (1, 5), (ii) methylmalonic aciduria (MMA) associated with reduced AdoCbl production and MUT deficiency (6, 7), or (iii) a combined HC and MMA defect (HC+MMA) (4). MMACHC and MMADHC are two proteins involved in the early Cbl processing steps that are shared by both mitochondrial and cytosolic targeting routes. *MMACHC* gene mutations are the most common cause of Cbl metabolic disorders, resulting in HC+MMA (8). Genetic defects of *MMADHC*, by contrast, uniquely cause any of the above three phenotypes (9, 10).

A possible explanation for the heterogeneous *MMADHC* phenotypes is the presence of distinct functional domains at the MMADHC protein level, responsible for trafficking to either mitochondrial MUT (of AdoCbl) or cytosolic MS (of MeCbl), or both. This hypothesis is supported by analysis of metabolic phenotypes from known disease mutations (11) and alanine-scanning mutagenesis (12). These studies dissect the 296-amino acid (aa) human MMADHC polypeptide functionally into the N-terminal 115 aa that is required for the mitochondrial route but dispensable for cytosolic trafficking, the C-terminal 180 aa that contribute to both routes, and two regions therein (aa 197–226 and 246–259) that have greater influence on the cytosolic than the mitochondrial route. Current knowledge points to a role of MMADHC in regulating Cbl delivery at the branch point between the mitochondrial and cytoplasmic target enzymes, albeit via an incompletely understood mechanism.

Furthermore, despite the *MMADHC* gene discovery seven years ago (10), few of the biochemical properties and functions of the MMADHC protein are known. Although previously postulated to contain Cbl and ATP binding motifs (10), MMADHC has been shown not to bind Cbl (13) or to hydrolyze ATP (14). Further biochemical exploration has been hampered in part by a lack of structural knowledge or sequence homologues. The inability of MMADHC to bind Cbl has implicated protein-protein interaction as the means of its Cbl-targeting role. This is supported by recent evidence from phage display (13, 15), bacterial two-hybrid studies (15), native PAGE (14), and surface plasmon resonance (13, 15) that MMADHC interacts directly with MMACHC *in vitro* (13, 15), and the interaction does not require the N-terminal 115 aa of MMADHC (14). Because MMACHC has been shown structurally (16, 17) and biochemically (18, 19) to bind various Cbl forms, and to process the upper Cbl ligand by reductive decyanation (19) or dealkylation (20) using the flavin cofactors FMN/FAD and GSH, respectively, it seems plausible that MMACHC-bound Cbl can partner with MMADHC to be ferried to the two destination enzymes.

To evaluate the importance and molecular basis of the MMACHC-MMADHC interaction, we have defined a minimal MMACHC interaction module of MMADHC, and demonstrated that experimental and patient missense mutations disrupt this interaction. We have further determined the structure of this interaction module from mouse MMADHC to 2.2 Å resolution, and established the 1:1 stoichiometry of the MMACHC-MMADHC heterodimer using small angle x-ray scattering (SAXS). Finally, based on the combined findings, we propose the first structural model of the MMACHC-MMADHC interaction.

## Experimental Procedures

### 

#### 

##### Recombinant Production of MMACHC and MMADHC

DNA fragments encoding human (*Homo sapiens*, IMAGE clone: 3826071) and mouse (*Mus musculus*, IMAGE clone: 3493526) MMADHC, harboring different N- and C-terminal boundaries, were amplified and subcloned into pNIC28-Bsa4 vector (GenBank^TM^ accession number EF198106) in-frame with a tobacco etch virus protease cleavable N-terminal His_6_ tag. Constructs of human *Hs*MMACHC in pNIC28-Bsa4 vector were prepared previously (16, 21). Site-directed mutations were constructed using the QuikChange mutagenesis kit (Stratagene) and confirmed by sequencing. Cloning and site-directed mutagenesis primers are available upon request. Proteins were expressed in *Escherichia coli* BL21(DE3)R3 and purified by affinity (Ni-Sepharose; GE Healthcare) and size-exclusion (Superdex 200; GE Healthcare) chromatography. For crystallization, MMADHC proteins were further purified by ion exchange chromatography (Resource Q; GE Healthcare). Selenomethionine (SeMet)-derivatized proteins were expressed using SelenoMethionine Medium Complete (Molecular Dimensions) and purified as above.

##### Crystallization and Structural Determination

Purified SeMet-derivatized and native *Mm*MMADHC_Δ128_ were concentrated to 15–20 mg/ml, and crystals were grown by sitting drop vapor diffusion at 4 °C. The mother liquor conditions are summarized in Table 1. Crystals were cryo-protected in mother liquor containing ethylene glycol (25% v/v) and flash-cooled in liquid nitrogen. X-ray diffraction data were collected at the Diamond Light Source beamline I04 and processed using XIA2. Selenium atoms were located using SHELXC/D (22) for initial phase calculation in SHELXE, and subsequently for automated building with BUCCANEER (23). The structure was solved by selenium single-wavelength anomalous diffraction phasing and refined using PHENIX (24), followed by manual rebuilding in COOT (25). The SeMet MMADHC model was used to solve the native structure by molecular replacement using PHASER (26). The final refined model consists of aa 132–157, 169–236, and 246–296 of *Mm*MMADHC. Atomic coordinates and structure factors for *Mm*MMADHC_Δ128_ have been deposited in the Protein Data Bank with the accession code 5A4R. Data collection and refinement statistics are summarized in Table 1.

##### Small Angle X-ray Scattering

For SAXS, MMACHC and MMADHC were cleaved by tobacco etch virus protease to remove the N-terminal His_6_ tag, followed by reverse affinity purification. Purified proteins were then incubated separately or together with the appropriate ligands and further purified by size-exclusion chromatography. Concentrations of proteins used were 24 mg/ml (MMADHC), 18 mg/ml (MMACHC), and 17 mg/ml (complex). Scattering data were collected at the Diamond Light Source B21 beamline. Data collection was performed either in-line with size-exclusion chromatography (KW404 column, Shodex) or in batch mode where the peak fractions were collected and immediately subjected to SAXS by flowing sample through an in-vacuum quartz capillary of 1.6-mm diameter. Data were collected using a Pilatus 2M detector (DECTRIS, Baden, Switzerland) at a sample-detector distance of 3914 mm and a wavelength of λ = 1 Å. The range of momentum transfer of 0.1 < *s* < 5 nm^−1^ was covered (*s* = 4πsinθ/λ, where θ is the scattering angle). For the in-line mode, 1-s exposures were collected, whereas for batch samples, a comparison of eighteen 10-s exposures was performed. Radiation damage was checked for the batch mode by monitoring changes in radius of gyration in each frame, where no significant changes were observed. The data were radially averaged, and the scattering of the buffer was subtracted. The forward scattering *I*(0), radius of gyration *R_g_*, pair distribution of the particle P(r), and maximum dimension *D*_max_ were analyzed using Scatter (27) and the ATSAS suite of programs (28).

##### Solution Characterization of MMACHC and MMADHC

Blue native-PAGE was performed with 25 μm protein (MMACHC and/or MMADHC) alone or in the presence of 50 μm cobalamin (MeCbl, AdoCbl, CNCbl) and/or 8 mm ligand (GSH, FMN, FAD), which was loaded onto the native-PAGE gel system, after preincubation in the dark at room temperature for 1 h, and then run following the manufacturer's instructions (Life Technologies). All blue native-PAGE experiments were performed at least twice independently. Analytical gel filtration (16), FMN/FAD binding by intrinsic fluorescence quenching (17), and differential scanning fluorimetry (21, 29), were performed as described previously. For native mass spectrometry, 75 μl of sample containing ∼4 mg/ml MMADHC and MMACHC incubated in the presence of 0.5 mm MeCbl and 2 mm reduced GSH underwent four desalting steps using Micro Bio-Spin columns (Bio-Rad) that were pre-equilibrated with 50 mm ammonium acetate buffer (pH 6.5). Following desalting, samples were loaded into a 1.0-ml gas-tight positive displacement syringe (Hamilton) that was inserted into the syringe pump. Samples were then directly infused with a constant flow rate of 6 μl/min through a PEEK capillary tubing (inner diameter, 0.005 inches) into a Q-TOF 6530 mass spectrometer attached to a standard electrospray ionization source (Agilent Technologies). The mass spectrometer was operated in positive ion mode, using the 1-GHz detector mode with a scan range of 100–20,000 *m/z* and a fragmentor voltage of 430 V. Following data acquisition, results were evaluated by using the MassHunter Qualitative Analysis software (Agilent Technologies).

## Results

### 

#### 

##### MMADHC aa 154–296 Is an MMACHC Interaction Module

**FIGURE 1.**
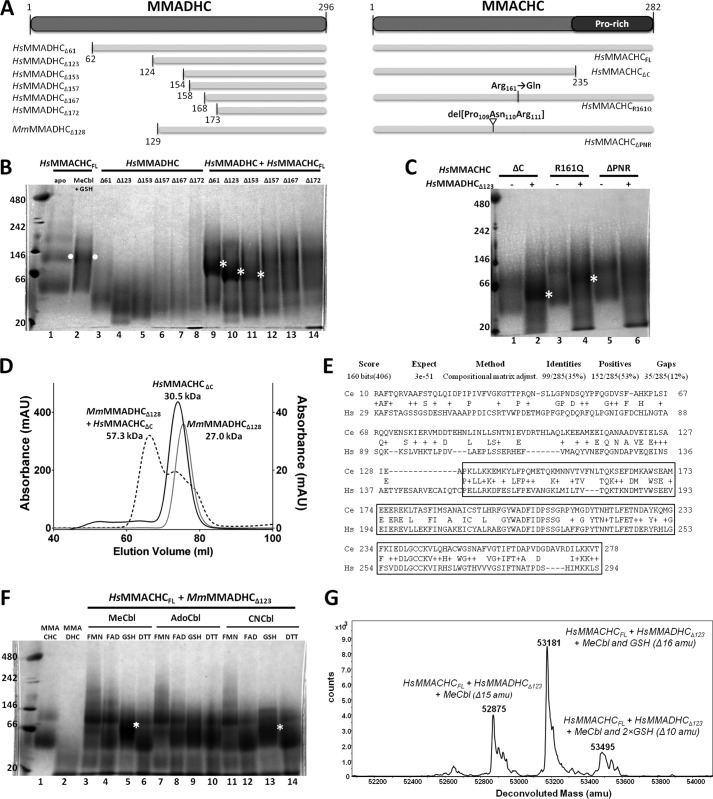
**Interaction study of MMACHC and MMADHC.** *A*, domain diagram showing recombinant human MMACHC and MMADHC constructs described in this figure. For reference, mouse construct *Mm*MMADHC_Δ128_ used to generate the crystal structure is also shown. *B*, interaction of human MMACHC full-length and MMADHC truncated proteins by BN-PAGE. *Lanes 1–8*: *single protein* controls including MMACHC_FL_ alone (*lane 1*) and with MeCbl and GSH (*lane 2*); and MMADHC truncations including MMADHC_Δ61_ (*lane 3*), MMADHC_Δ123_ (*lane 4*), MMADHC_Δ153_ (*lane 5*), MMADHC_Δ157_ (*lane 6*), MMADHC_Δ167_ (*lane 7*), and MMADHC_Δ172_ (*lane 8*). *Lanes 9–14*: *combined protein* incubation of MMACHC_FL_ in presence of MeCbl and GSH with MMADHC_Δ61_ (*lane 9*), MMADHC_Δ123_ (*lane 10*), MMADHC_Δ153_ (*lane 11*), MMADHC_Δ157_ (*lane 12*), MMADHC_Δ167_ (*lane 13*), and MMADHC_Δ172_ (*lane 14*). *C*, interaction of MMACHC mutants and MMADHC_Δ123_ by BN-PAGE, in the presence of MeCbl and GSH. *Lanes 1–6*: MMACHC_ΔC_ alone (*lane 1*) and with MMADHC_Δ123_ (*lane 2*); MMACHC_R161Q_ alone (*lane 3*) and with MMADHC_Δ123_ (*lane 4*); and MMACHC_ΔPNR_ alone (*lane 5*) and with MMADHC_Δ123_ (*lane 6*). For *B* and *C*, *white dots* indicate MMACHC homodimer, and *white asterisks* indicate MMACHC-MMADHC heterodimer. *D*, analysis of complex formation by size-exclusion chromatography including chromatographs of *Mm*MMADHC_Δ123_ (*gray line*), *Hs*MMACHC_ΔC_ + MeCbl + GSH (*black line*), and *Mm*MMADHC_Δ123_ + *Hs*MMACHC_ΔC_ + MeCbl + GSH (*black dotted line*). *x* axis: elution volume; *left y* axis: absorbance (milliabsorbance units (*mAU*)) for *Hs*MMACHC_ΔC_ + MeCbl + GSH; right *y* axis: absorbance (mAU) for *Mm*MMADHC_Δ123_ and *Mm*MMADHC_Δ123_ + *Hs*MMACHC_ΔC_ + MeCbl + GSH. Molecular weights of each peak are calculated from a calibration curve using molecular weight protein standards (Sigma-Aldrich). *E*, sequence alignment of MMADHC from *H. sapiens* (*HS*, NP_056517.1, human) and predicted MMADHC protein (Y76A2B.5) from *C. elegans* (*Ce*) using the BLAST server (http://blast.ncbi.nlm.nih.gov/Blast.cgi). The *boxed region* represents the smallest interacting construct of *Hs*MMADHC for *Hs*MMACHC. *F*, BN-PAGE of *Hs*MMACHC_FL_ and *Hs*MMADHC_Δ123_ under various conditions. *White asterisks* indicate MMACHC-MMADHC heterodimer. *G*, native mass spectrometry of *Hs*MMACHC_FL_ and *Hs*MMADHC_Δ123_ incubated with MeCbl and GSH. Expected sizes: *Hs*MMACHC_FL_, 31818.6 Da; *Hs*MMADHC_Δ123_, 19727.7 Da; MeCbl, 1343.6 Da; and GSH, 307.3 Da.

To better understand the required regions, ligand dependence, and stoichiometry of their *in vitro* interactions, we generated a series of human (*Hs*) MMADHC and MMACHC truncation proteins by recombinant expression (Fig. 1*A*). Using blue native-PAGE (BN-PAGE) (Fig. 1*B*), full-length MMACHC (*Hs*MMACHC_FL_) alone migrated as two bands (*lane 1*), consistent with its known monomeric and dimeric forms, whereas the dimeric band became more prominent in the presence of MeCbl and GSH (*lane 2*, *white dot*). The *Hs*MMADHC proteins with various N-terminal truncations migrated as single bands (*lanes 3–8*) corresponding to their respective monomers, corroborating previous investigations (13). Complex formation, as judged by the appearance of an additional band in BN-PAGE as compared with single-protein controls, was observed when *Hs*MMACHC_FL_ preincubated with MeCbl and GSH was added to *Hs*MMADHC_Δ61_ or *Hs*MMADHC_Δ123_, or to a lesser extent, *Hs*MMADHC_Δ153_ (*lanes 9–11*, *white asterisk*). However,no complex band was observed with N-terminal truncation of *Hs*MMADHC beyond aa 154 (*Hs*MMADHC_Δ157_, *Hs*MMADHC_Δ167_, or *Hs*MMADHC_Δ172_; *lanes 12–14*) under the same conditions. The *Hs*MMADHC-*Hs*MMACHC complex bands migrated at a position intermediate of the estimated monomeric and dimeric *Hs*MMACHC bands, suggesting a possible 1:1 heterodimeric complex. *Hs*MMACHC_ΔC_, which lacks the C-terminal Pro-rich region (aa 236–282; Fig. 1*A*), was sufficient for complex formation with *Hs*MMADHC_Δ123_ (Fig. 1*C*, *lane 2*). Size-exclusion chromatography using *Mm*MMADHC_Δ128_ and *Hs*MMACHC_ΔC_ proteins further confirmed the regions required for interaction and the 1:1 stoichiometry of the complex (Fig. 1*D*). Therefore, the MMADHC region C-terminal to aa 154 and the MMACHC region without the Pro-rich C terminus is sufficient for direct protein-protein interaction. Notably, the MMADHC C-terminal region is the most evolutionarily conserved section of the polypeptide from human to *Caenorhabditis elegans* (Fig. 1*E*), suggesting selected retention of this interaction module in evolution.

In agreement with previous work (14), complex formation is favored by preincubation of proteins with GSH and MeCbl, conditions known to facilitate upper Cbl ligand (R-group) removal. We observed either significantly weaker or no interaction between *Hs*MMACHC_FL_ and *Hs*MMADHC_Δ123_ when GSH was replaced by other reducing factors (FMN, FAD, DTT) or when MeCbl was replaced by other Cbl forms (AdoCbl, CNCbl) (Fig. 1*F*), conditions known to result in much slower (>10-fold) or no R-group removal (30, 31). We further investigated two *Hs*MMACHC mutants with reduced MeCbl dealkylation activity (*Hs*MMACHC_ΔPNR_, missing aa 109–111 (16), or *Hs*MMACHC_R161Q_ (16, 32)), on their ability to complex with *Hs*MMADHC_Δ123_. Upon incubation with MeCbl and GSH, the *Hs*MMACHC_R161Q_ mutant was less able than *Hs*MMACHC_FL_ to complex with *Hs*MMADHC_Δ123_ (compare Fig. 1*C*, *lane 4* with Fig. 1*B*, *lane 10*), whereas *Hs*MMACHC_ΔPNR_ did not complex with *Hs*MMADHC_Δ123_ at all (Fig. 1*C*, *lane 6*).

The MMACHC-MMADHC interaction is further studied by native mass spectrometry, where the complex of *Hs*MMACHC_FL_ and *Hs*MMADHC_Δ123_, incubated in the presence of MeCbl and GSH, generated two peaks consistent with a 1:1 stoichiometry, one missing the 15 atomic mass units of the methyl group (Fig. 1*G*, 52,875 atomic mass units) and the other missing 16 atomic mass units, corresponding to the methyl group plus the hydrogen from GSH (Fig. 1*G*, 53,181 atomic mass units). Thus, R-group removal appears to be a prerequisite for MMACHC-MMADHC interaction.

##### MMADHC Contains a Nitroreductase-like Fold with Homology to MMACHC

**FIGURE 2.**
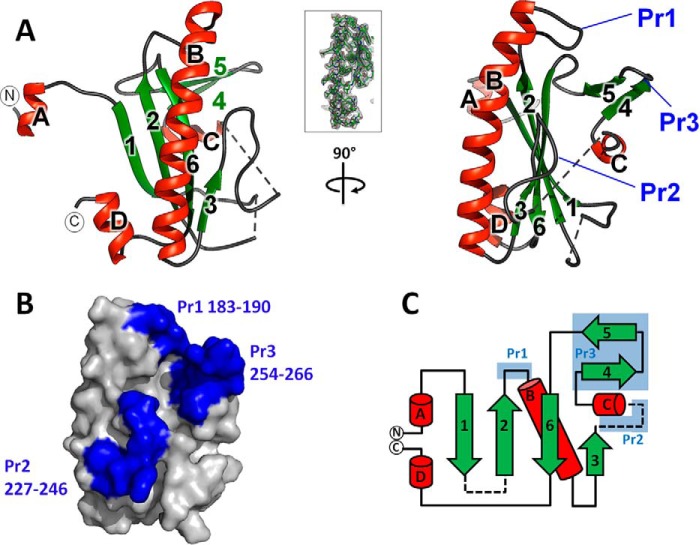
**Structure of the MMACHC interaction module of MMADHC.** *A*, graphic representation of the *Mm*MMADHC_Δ128_ structure in orthogonal views. Secondary structures are colored *green* for β-sheets and *red* for α-helices. The first (aa 132) and last (aa 296) residues observed in the structure are labeled with *N* and *C*, respectively. *Dotted lines* indicate disordered regions. *Inset*: view of the σ-weighted (2*F_o_* − *F_c_*) electron density map of *Mm*MMADHC_Δ128_ aa region 190–217, contoured at σ = 1. *B*, surface representation of *Mm*MMADHC_Δ128_ (same orientation as *A*, *right panel*) highlighting the three protrusions (Pr1–Pr3) in *blue. C*, topology diagram of the *Mm*MMADHC_Δ128_ secondary structure with the same coloring and labeling as in *A* and *B*. Disordered regions are shown as *dashed lines*.

We next characterized MMADHC by x-ray crystallography to elucidate the molecular properties of its MMACHC interaction module. To maximize the probability of crystallization, we adopted a cross-species approach that involved the study of recombinant human, *M. musculus*, and *Xenopus laevis* MMADHC proteins, coupled with an extensive survey of construct boundaries. From >40 purified proteins, we successfully crystallized *M. musculus* MMADHC aa 129–296 (*Mm*MMADHC_Δ128_; Figs. 1*A* and 2A), a region bearing 93% identity with the human sequence. In our BN-PAGE assay, *Mm*MMADHC(*e.g. Mm*MMADHC_Δ128_) can substitute the equivalent human construct in stable complex formation with *Hs*MMACHC (not shown). Despite repeated attempts, the equivalent *Hs*MMADHC construct did not crystallize. The structure of *Mm*MMADHC_Δ128_ (Fig. 2, *A* and *B*), determined to 2.6 Å resolution by single-wavelength anomalous dispersion phasing for the selenomethionine (SeMet) derivative, and to 2.2 Å by molecular replacement for the native protein (Table 1), is a mixed α/β domain composed of a central four-stranded antiparallel β-sheet (β1, β2, β6, β3) flanked by a 14-turn helix (αB) and a short β-turn (β4-β5) (Fig. 2*C*).

**TABLE 1:** **Data collection and refinement statistics for *Mm*MMADHC structure**

	*Mm*MMADHC_Δ128_ SeMet*^a^*	*Mm*MMADHC_Δ128_ native*^b^*
**Data collection and processing***^c^*		
Beamline	Diamond I04	Diamond I04
Wavelength (Å)	0.9782	0.9782
Unit cell parameters a, b, c (Å)	73.5 88.7 65.7	74.2 89.5 64.7
Unit cell parameters α, β, γ (°)	90.00 90.00 90.00	90.00 90.00 90.00
Space group	C 2 2 21	C 2 2 21
Resolution range (Å)	44.33–2.61 (2.68–2.61)	44.76–2.25 (2.31–2.25)
Observed/Unique reflections	78,726/6293 (6117/471)	51,530/10,530 (3778/762)
*R*_sym_ (%)	12.2 (54.8)	2.5 (59.8)
CC1/2	0.99 (0.96)	1.0 (0.80)
*I*/σ(*I*)	15.1 (4.7)	31.6 (2.6)
Completeness (%)	Anomalous 92.1 (98.8)	99.8 (99.6)
Multiplicity	Anomalous 6.7 (6.8)	4.9 (5.0)

**Refinement**		
*R*_cryst_ (%)		22.9
*R*_free_ (%)		25.5
Wilson *B* factor (Å^2^)		55.5
Average total *B* factor (Å^2^)		66.2
r.m.s.d. bond length (Å^2^)		0.0034
r.m.s.d. bond angle (°)		0.87
Missing residues		129–131, 160–168, 237–245

**MolProbity analysis**		
Clashscore		5.98 (98th percentile)
Ramachandran favored (%)		96.48%
Ramachandran disallowed (%)		0.00%
Rotamer outliers (%)		0.00%

**PDB code**		5A4R

*^a^* Crystallization condition: 28% PEG3350, 100 mm Bis-Tris, pH 6.5, 250 mm NaCl.

*^b^* Crystallization condition: 22% polyacrylic acid 5100, 100 mm HEPES, pH 7.5, 20 mm MgCl_2_.

*^c^* Data from highest resolution shell are shown in parentheses.

**FIGURE 3.**
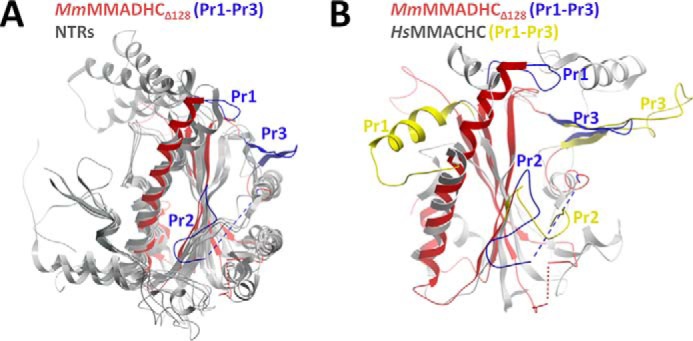
**Structural comparison of MMADHC and proteins of the nitroreductase family.** *A*, structural superposition of *Mm*MMADHC_Δ128_ (*red*) and two other NTRs (Protein Data Bank (PDB): 2HAY, 2ISL; *gray*). *B*, superposition of *Mm*MMADHC_Δ128_ (*red*) and MMACHC (PDB: 3SOM; *gray*), highlighting the absence of the *Hs*MMACHC four-helix cap domain (aa 185–234) in *Mm*MMADHC_Δ128_ as well as the different orientations of protrusions Pr1 and Pr2 (*Hs*MMACHC: Pr1 aa 69–77; Pr2 aa 104–116; *Mm*MMADHC_Δ128_: Pr1 aa 183–190, Pr2 aa 227–246) between the proteins. Protrusions (Pr1–Pr3) are colored *blue* for *Mm*MMADHC_Δ128_ and *yellow* for *Hs*MMACHC.

**FIGURE 4.**
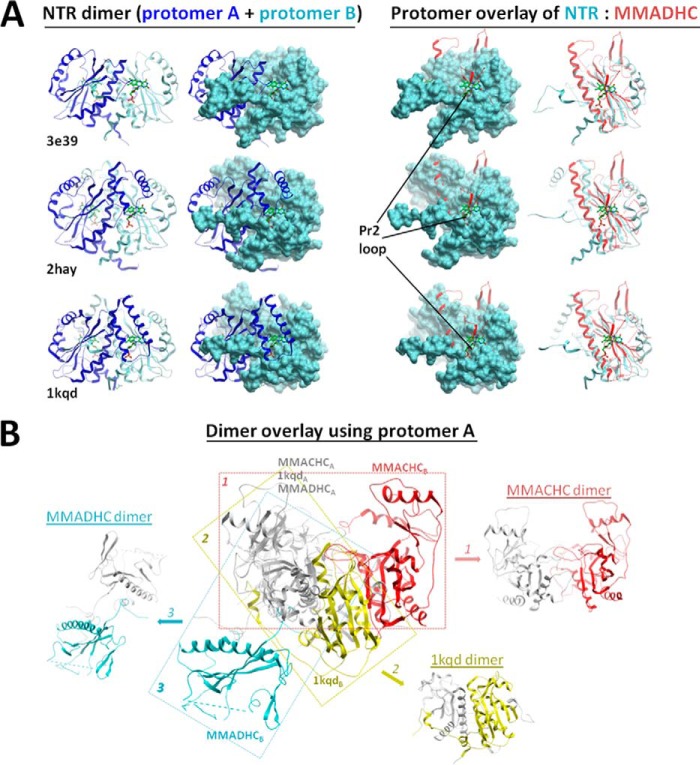
**Comparison of MMADHC to other NTR dimers.** *A*, overlay of the *Mm*MMADHC_Δ128_ structure (*red*) with nitroreductases from *Desulfovibrio desulfuricans* (PDB: 3E39), *Streptococcus pyogenes* (PDB: 2HAY), and *Enterobacter cloacae* (PDB: 1KQD). The canonical FMN/FAD binding site of NTRs (*left*, indicated by the *green stick ligand*), located at the conventional dimer interface, is not present in MMADHC (*right*) due to disruption by its protrusion loop Pr2. *B*, structural alignment of a single subunit (protomer A) of *Mm*MMADHC_Δ128_ (MMADHC), *Hs*MMACHC_FL_ (MMACHC, PDB: 3SOM), and an NTR (PDB: 1KQD) in *gray*, with the second subunit (protomer B) of their crystallographic dimers color-coded (MMADHC in *blue*; NTR in *yellow*; MMACHC in *red*), demonstrating the lack of a conserved dimeric orientation and interface among the three structures.

A structural comparison of *Mm*MMADHC_Δ128_ with other proteins using DALI (33) revealed that the core structure is derived from the nitroreductase (NTR) fold (Fig. 3*A*), primarily found in flavoenzymes and oxidoreductases that utilize FMN/FAD cofactors and form homodimers by β-strand exchange, as well as MMACHC (Fig. 3*B*). Nevertheless, *Mm*MMADHC_Δ128_ has extensively modified the NTR fold with three loop protrusions (Pr1–Pr3), rendering it functionally different from classical NTRs. Firstly, *Mm*MMADHC_Δ128_ does not bind FMN/FAD in solution (data not shown) because the canonical binding site found in NTRs is disrupted by part of the MMADHC Pr2 loop protrusion (Fig. 4*A*). Secondly, although *in crystallo Mm*MMADHC_Δ128_ reveals a symmetry-related dimer, its dimeric interface does not match that of the conventional NTRs (Fig. 4*B*). Indeed, *Mm*MMADHC_Δ128_ is a monomer in solution by BN-PAGE, size-exclusion chromatography (not shown), and SAXS (see “A Structural Model of the MMACHC-MMADHC Complex”), consistent with *Hs*MMADHC constructs migrating as monomers in BN-PAGE (Fig. 1*B*).

To our surprise, the closest structural homologue of MMADHC is its interaction partner MMACHC (DALI (33) Z-score, 5.2), despite a lack of obvious sequence conservation. Like MMADHC, MMACHC contains three loop protrusions, at equivalent spatial positions, in the core NTR fold (16, 17) (Fig. 3*B*). MMACHC also lacks the classical FMN binding site present among NTRs, while retaining the ability to bind flavins in solution (17), likely involving a non-canonical binding site (16). The MMADHC and MMACHC structures superimpose only moderately and within the core NTR secondary structure elements (C^α^-r.m.s.d. 3.0 Å, 96 aligned aa), beyond which they differ substantially (Fig. 3*B*). For example, *Mm*MMADHC_Δ128_ does not contain the four-helix cap domain and differs in length and conformation of two protrusions as compared with MMACHC, all of which form part of the MMACHC Cbl binding pocket (16, 17). These extensive structural differences from MMACHC likely account for the reported lack of Cbl binding capability of MMADHC (13). We additionally found no sequence elements or homology consistent with ATPase activity.

##### Patient and Experimental MMADHC Mutations Knock Out Interaction with MMACHC

**FIGURE 5.**
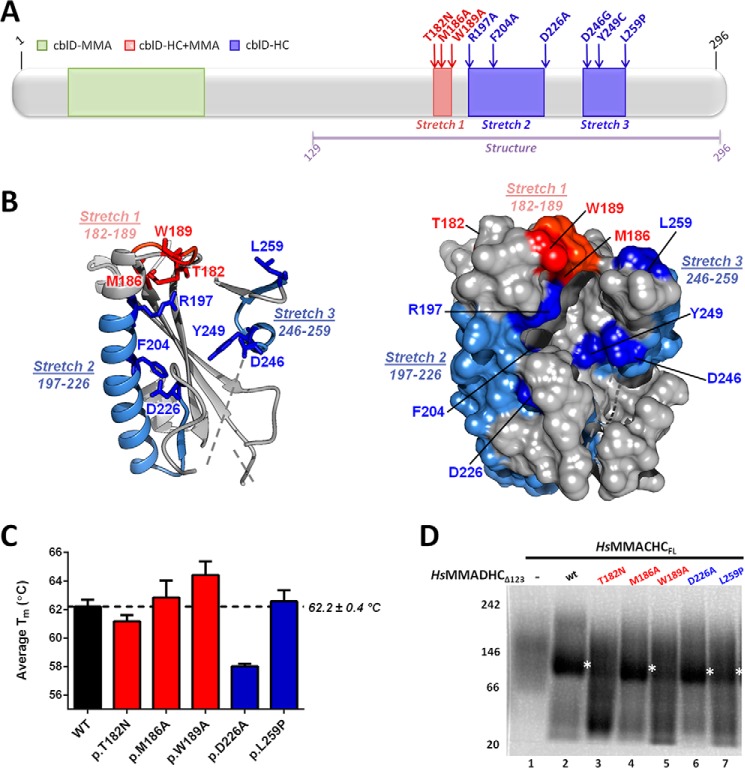
**Structural and biochemical analysis of MMADHC missense mutations.** *A*, domain diagram of MMADHC, with missense mutations color-coded according to their cellular defects (10–12), namely MMA (*green*), combined HC+MMA (stretch 1, *red*), or HC (stretches 2 and 3, *blue*) phenotypes. *Purple line* indicates aa region observed in the crystal structure. *B*, graphic (*left*) and surface (*right*) representations of *Mm*MMADHC_Δ128_ mapped with stretches 1–3 and individual mutations found within these regions. *Dotted lines* indicate disordered regions. *C*, analysis of thermal unfolding curves of various protein constructs reveals a melting temperature (*T_m_*) of 62 °C (*right*) for *Hs*MMADHC_Δ123_, which is relatively unchanged for all mutants with the exception of *Hs*MMADHC_Δ123-D226A_ where the *T_m_* is slightly decreased as compared with wild type. *Error bars* indicate means ± S.E. *D*, interaction of *Hs*MMACHC_FL_ with wild-type or mutant *Hs*MMADHC_Δ123_ studied by BN-PAGE. Depicted are: *Hs*MMACHC_FL_ with MeCbl and GSH alone (*lane 1*), and in combination with *Hs*MMADHC_Δ123_ (*lane 2*), *Hs*MMADHC_Δ123-T182N_ (*lane 3*), *Hs*MMADHC_Δ123-M186A_ (*lane 4*), *Hs*MMADHC_Δ123-W189A_ (*lane 5*), *Hs*MMADHC_Δ123-D226A_ (*lane 6*), and *Hs*MMADHC_Δ123-L259P_ (*lane 7*). *White asterisks* indicate the MMACHC-MMADHC heterodimer.

Our previous mutagenesis studies in patient fibroblasts (11, 12) revealed specific MMADHC regions responsible for the intracellular targeting of the two cobalamin cofactors. These mutations correlate with the MMA, HC, or HC+MMA phenotypes based on their amino acid location (Fig. 5*A*). Missense mutations causing decreased MeCbl production (HC phenotype) or both AdoCbl and MeCbl production (HC+MMA phenotype) in cells are found within three sequence stretches at the MMADHC C-terminal half (Fig. 5*A*, *stretches 1–3*). When mapped onto our *Mm*MMADHC_Δ128_ structure, these three stretches cluster in spatial proximity and are surface-accessible (Fig. 5*B*). We investigated whether these mutations impact on the MMACHC-MMADHC interaction by introducing onto the *Hs*MMADHC_Δ123_ construct a selection of HC and HC+MMA causing mutations from the three stretches (Stretch 1: p.T182N, p.M186A, p.W189A; Stretch 2: p.D226A; Stretch 3: p.L259P). The recombinant mutant proteins behaved similarly to wild type in terms of expression level, protein solubility (not shown), and thermal stability (Fig. 5*C*), where only p.D226A led to slightly unstable protein. We found interaction of *Hs*MMACHC_FL_ (incubated with MeCbl and GSH) with *Hs*MMADHC_Δ123-T182N_ and *Hs*MMADHC_Δ123-W189A_ to be severely decreased (Fig. 5*D*, *lanes 3* and *5*), but retainedfor *Hs*MMADHC_Δ123-M186A_, *Hs*MMADHC_Δ123-D226A_, and *Hs*MMADHC_Δ123-L259P_ (Fig. 5*D*, *lanes 4*, *6*, and *7*). These findings suggest that residues within Stretch 1 (Thr-182, Trp-189) occur at the binding interface with MMACHC.

##### A Structural Model of the MMACHC-MMADHC Complex

**FIGURE 6.**
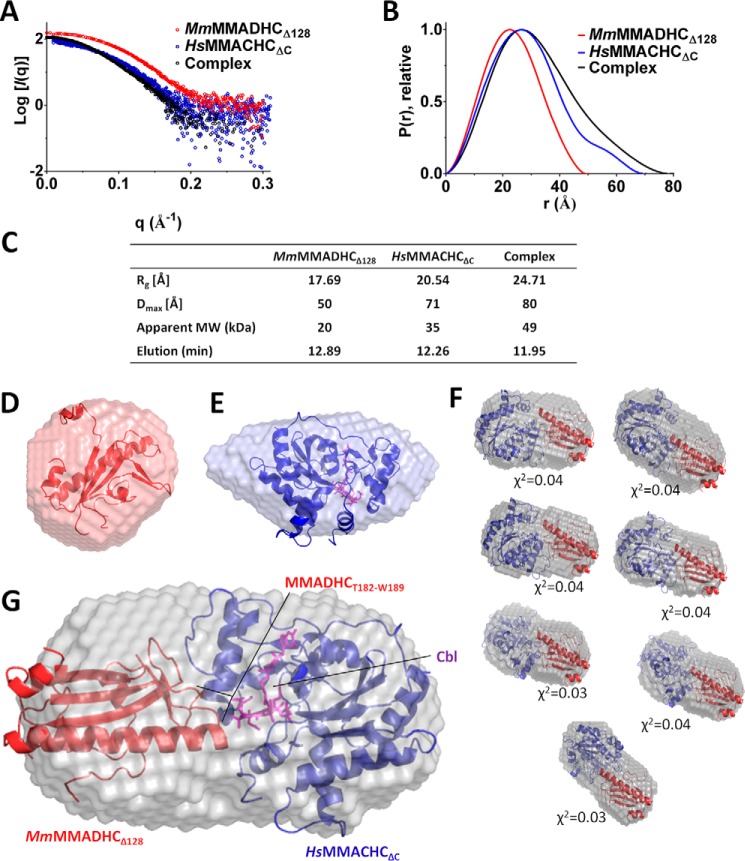
**SAXS analysis of the MMACHC-MMADHC interaction.** *A*, raw scattering curves for *Mm*MMADHC_Δ128_ (*red*), *Hs*MMACHC_ΔC_ (*blue*), and their complex (*black*). *B*, P(r) plots, calculated with Scatter (27) and normalized to peak height, show differences between the single proteins and the complex. Guinier plots indicate aggregation-free data. *C*, table of radius of gyration (*R_g_*), maximal intraparticle dimension (*D*_max_) as calculated from the Guinier plot, apparent molecular weight (*MW*), and elution time from the in-line HPLC column. *D* and *E*, *ab initio* bead models calculated with 13 runs of DAMMIF (39) for *Mm*MMADHC_Δ128_ fitted with its x-ray structure (this study) (*D*) and *Hs*MMACHC_ΔC_ fitted with its x-ray structure (PDB: 3SOM) (*E*). *F*, top seven models of the *Hs*MMACHC_ΔC_-*Mm*MMADHC_Δ128_ complex produced by ZDOCK with fitted χ^2^ values shown. The SAXS envelope is overlaid with modeled orientations of *Hs*MMACHC_ΔC_ (*blue*) and *Mm*MMADHC_Δ128_ (*red*) structures. *Mm*MMADHC_Δ128_ is presented in the same orientation on all models for better comparison. *G*, *Hs*MMACHC_ΔC_-*Mm*MMADHC_Δ128_ complex fitted with a representative example of the ZDOCK (35) complex model. In *E* and *G*, Cbl is shown as *purple sticks*.

We next utilized SAXS, a low-resolution structural characterization method in solution (34), to construct a model of the MMACHC-MMADHC interaction. To ensure that the SAXS data most closely resembled the ordered aa regions revealed from the MMACHC and MMADHC structures, we reconstituted a complex from untagged *Mm*MMADHC_Δ128_ and untagged *Hs*MMACHC_ΔC_ proteins in the presence of MeCbl and GSH. The Guinier approximation and pair distribution function P(r) calculated from the scattering data (Fig. 6, *A* and *B*) suggest a radius of gyration (*R_g_*) of 24.7 Å and maximal intraparticle dimension (*D*_max_) of 80 Å for the complex, both parameters larger than those from either protein component alone (Fig. 6*C*). Further, the elution time of the protein complex from the in-line HPLC column was earlier than either protein alone, giving rise to a calculated molecular weight consistent with a 1:1 stoichiometry (Fig. 6*C*). The *ab initio* envelope of the *Mm*MMADHC_Δ128_-alone sample fits well to one monomer (Fig. 6*D*). MMACHC is known to exist in a monomer:dimer equilibrium (Fig. 1) (16), and the monomeric species selected for SAXS analysis is consistent with one protomer in the *ab initio* envelope (Fig. 6*E*). However, for the MMACHC-MMADHC complex, the *ab initio* envelope (Fig. 6*F*) gives an elongated dimension of 80, 40, and 40 Å. One protomer from each of *Hs*MMACHC_ΔC_ and *Mm*MMADHC_Δ128_ would account for all the mass of the envelope, further indicating a 1:1 heterodimeric stoichiometry that agrees with our BN-PAGE, size-exclusion chromatography, and native mass spectrometry data (Fig. 1). Although the relative orientation of both proteins cannot be ascertained by SAXS, we applied our mutagenesis and interaction data (Fig. 5) to direct protein docking of the complex using the ZDOCK server (35), given that residue Thr-182 of *Mm*MMADHC should be involved in the interaction. With this constraint, the top seven models generated from ZDOCK (Fig. 6*F*) all yield a good fit (χ^2^ ∼0.03–0.04) to the measured scattering. These models are consistent with an MMACHC-MMADHC interface that involves the Cbl binding region of MMACHC and aa 182–189 (Fig. 5, *stretch 1*) of MMADHC (Fig. 6*G*). A similar docking run was performed with HADDOCK (36), yielding consistent models (data not shown).

## Discussion

The role of MMACHC within the early stages of the Cbl processing pathway has previously been clarified from the available structural and biochemical evidence of its Cbl binding and processing activities (16–20). By contrast, the role of MMADHC, tasked with directing Cbl to the destination enzymes yet unable to bind Cbl itself, is much less clear. This study presents the first structural characterization of MMADHC to provide novel insights into its unknown function, with the key findings that: (i) MMADHC functions by binding Cbl-laden MMACHC, but only post-Cbl processing, *i.e.* after the upper axial ligand of Cbl has been removed; (ii) the MMACHC interaction module of MMADHC contains an ingeniously modified NTR fold that abolishes homodimerization, favors heterodimerization with another modified NTR fold from MMACHC, and accounts for the reported lack of Cbl binding (13); and (iii) missense mutations of MMADHC that reduced both AdoCbl and MeCbl production in cells can be explained at least in part by an abrogated interaction with MMACHC.

**FIGURE 7.**
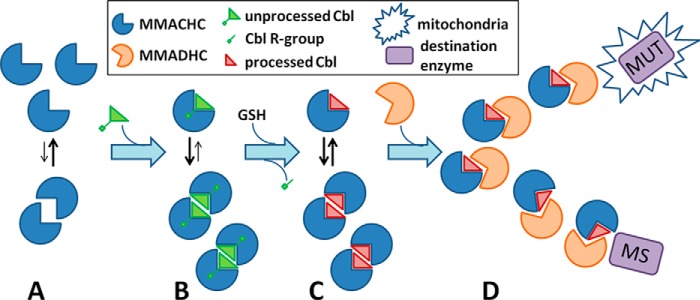
**Proposed role of the MMACHC-MMADHC complex in Cbl targeting.** *A*, MMACHC (*blue sector*) in the unbound state exists primarily as a monomer. *B*, upon binding unprocessed Cbl with an intact upper axial ligand (*triangle*), it can shift toward the homodimeric state. *C*, following processing of Cbl (*green* to *red triangle*) via GSH-mediated removal of its upper axial ligand (*green stick* and *square*), MMACHC is available for interaction with MMADHC (*orange sector*). *D*, MMADHC binds MMACHC in a 1:1 heterodimer and escorts Cbl-laden MMACHC either toward the mitochondria (*star*) for use by methylmalonyl-CoA MUT or to MS in the cytosol.

Our data illustrate the application of protein-protein interaction in the early Cbl-targeting steps of the pathway for both the mitochondrial (AdoCbl to MUT) and the cytosolic (MeCbl to MS) targeting routes. Such protein-protein interaction is essential to the targeting process, and is disrupted by a known disease mutation of MMACHC that precludes B_12_ processing and by mutations of MMADHC that alter the binding interface. We propose a mechanistic model for the MMACHC-MMADHC complex whereby the adaptation of the NTR fold by both proteins favors heterodimerization to form the “Cbl trafficking chaperone.” In this model, MMACHC in the Cbl-free form could exist in a monomer-homodimer equilibrium (Fig. 7*A*). The self-association of MMACHC into homodimer, likely with a high dissociation rate that precludes its isolation *in vitro* (13, 16, 17), is enriched in the presence of Cbl ligand (Fig. 7*B*) and may serve to close the enzymatic active site for proper Cbl processing (16) (Fig. 7*C*). Cbl-laden MMACHC (monomer/homodimer), with the upper axial ligand now removed, preferentially binds monomeric MMADHC as a 1:1 heterodimer rather than binding to itself, resulting in the “trafficking chaperone” that delivers processed Cbl to its target destinations (Fig. 7*D*).

Interestingly, only the C-terminal 154 aa of MMADHC is required for the interaction with MMACHC. As such, the exact function of the MMADHC N terminus remains to be determined. Predicted to be largely disordered, the N-terminal ∼115 aa harbors a mitochondrial targeting sequence (aa 1–12) and has been shown to be required for cellular AdoCbl production only (11). Thus, this protein region is likely only required for function within, or direction to, the mitochondria.

Because MMADHC mutations that abolished direct interaction with MMACHC result in the combined HC+MMA phenotype (11, 12), it is conceivable that disruption of the MMACHC-MMADHC complex will result in an inability to deliver Cbl to both destination enzymes MS and MUT. A possible role of MMACHC in the mitochondria, where it has not been detected either on its own or in complex (37), remains undetermined. The dysfunction caused by *MMADHC* missense mutations, which result in a loss of MS activity only, *i.e.* the so-called *cblD*-HC mutations (10), also remains unexplained. Because MMACHC has recently been shown to bind various isoforms of MS (38), the *cblD*-HC mutations could affect a larger, yet unidentified multi-protein complex, likely including at least MMACHC and MS, but possibly also methionine synthase reductase.

In conclusion, we have structurally characterized the MMACHC-interacting module of MMADHC and demonstrated that specific MMADHC mutations that cause combined loss of AdoCbl and MeCbl production in cells can interfere with MMACHC interaction. Further studies will clarify the affinity of these proteins for each other, and how this protein-protein interaction results in Cbl delivery in the cellular context.

## Author Contributions

B. F., D. S. F., M. R. B., and W. W. Y. conceived of the study. D. S. F., T. P., and V. F. cloned the MMADHC constructs. D. S. F., J. K., and F. F. purified, crystallized and determined structure of MMADHC. F. F. and J. K. reconstituted the complex and carried out SAXS analysis. M. S. and T. J. M. performed BN-PAGE. M. S. and R. C. performed and analyzed native mass spectrometry. D. S. F. and W. W. Y. wrote the manuscript with editing and proofreading support from all other co-authors.

## Footnotes

5 The abbreviations used are:

Cbl: cobalamin
MeCbl: methyl-Cbl
AdoCbl: adenosyl-Cbl
CNCbl: cyanocobalamin
MS: methionine synthase
MUT: methylmalonyl-CoA mutase
HC: homocystinuria
MMA: methylmalonic aciduria
aa: amino acid(s)
SAXS: small-angle x-ray scattering
NTR: nitroreductase
SeMet: selenomethionine
BN-PAGE: blue native-PAGE
Bis-Tris: 2-(bis(2-hydroxyethyl)amino)-2-(hydroxymethyl)propane-1,3-diol
r.m.s.d: root mean square deviation
FL: full-length
*Hs*: *Homo sapiens*
*Mm*: *Mus musculus*.

## Addendum

During the revision of this manuscript, Yamada *et al.* (*J. Biol. Chem.*, 10.1074/jbc.M115.682435 (40)) reported the human MMADHC structure of a region corresponding to our mouse structure, and consistent with our structural observations described here.
